# Time trends (1995–2008) in dietary habits among adolescents in relation to the Norwegian school fruit scheme: the HUNT study

**DOI:** 10.1186/s12937-019-0501-z

**Published:** 2019-11-20

**Authors:** Ingrid Marie Hovdenak, Elling Bere, Tonje Holte Stea

**Affiliations:** 1 Department of Public Health, Sport and Nutrition, University of Agder, Universitetsveien 25, 4630 Kristiansand, Norway; 2 Department of Health and Inequalities, Centre for Evaluation of Public Health Measures, Norwegian Institute of Public Health, Marcus Thranes Gate 6, 0473 Oslo, Norway

**Keywords:** Fruits, Vegetables, School fruit schemes, Adolescents, The young-HUNT study

## Abstract

**Introduction:**

The importance of healthy eating in adolescence is well established. The present study examined possible effects of the free Norwegian School Fruit Scheme (NSFS), changes in dietary habits between 1995 and 2008, and whether secular changes in dietary habits differed among schools who implemented the NSFS during September 2007.

**Method:**

We used data from the Young-HUNT1 survey conducted from 1995 to 1997 and the Young-HUNT3 survey conducted from 2006 to 2008, which are part of the Nord-Trøndelag Health Study (HUNT), a longitudinal population health study. To evaluate the NSFS, the date Young-HUNT3 participants answered the questionnaire was used to identify affiliation to the intervention group (post-September 2007, *n* = 1892) or control group (pre-September 2007, *n* = 2855). To explore dietary habits over time, adolescents attending the same schools in Young-HUNT1 (*n* = 4137) and Young-HUNT3 (*n* = 4113) were included. Further, we investigated secular changes in dietary habits according to school type (intervention schools vs control schools). In all analysis, we explored possible differential effects according to socioeconomic status (SES) and gender. A questionnaire measured adolescents’ consumption of fruit, vegetables, candy, potato chips, sugar-sweetened beverages (SSB) and artificially sweetened beverages (ASB). Educational intention was used as a proxy for SES. Multilevel logistic regression was used.

**Results:**

Within Young-HUNT3, the intervention group showed increased odds of daily consumption of fruit (aOR 1.7, 95% CI = 1.3–2.4) compared to the control group. Over time, adolescents were more likely to consume fruit (aOR = 1.48, 95% CI = 1.28–1.71), vegetables (OR = 1.41, 95% CI = 1.28–1.53), potato chips (aOR = 1.60, 95% CI = 1.26–2.04) and SSB (OR = 2.02, 95% CI = 1.66–2.45). Secular changes for fruit differed by school type: adolescents in intervention schools had higher odds of daily consumption (aOR = 1.82, 95% CI = 1.38–2.38) than those in control schools (aOR 1.26, 95% CI = 1.07–1.47).

**Conclusion:**

The results indicated that the NSFS increased adolescents’ fruit consumption. In the period assessed, the study identified positive and negative changes in adolescents’ dietary habits.

## Introduction

To improve overall health, a diet high in fruit and vegetables and low in sugar, fat and sodium is recommended by the World Health Organization and The Norwegian Directorate of Health [1–3]. High consumption of sugar-sweetened beverages (SSB) has been associated with weight gain [4], and low consumption of fruit and vegetables has been associated with increased risk of cardiovascular disease, type two diabetes and cancers [5]. As studies have indicated that food habits established in childhood may track into adulthood [6], a healthy diet rich in fruit and vegetables and low in SSB will most likely reduce future incidences of chronic disease.

In 2010, most adolescents in European countries had lower consumption of fruit and vegetables than recommended by international authorities. Daily fruit consumption ranged from 15 to 49% of adolescents, and vegetable consumption from 20 to 55% [1, 7]. A relatively high percentage of adolescents reported daily consumption of SSB [8, 9].

Although social inequalities in dietary habits are less evident among adolescents compared to adults [10], adolescents living in families with low socioeconomic status (SES) have reported less favourable diets, including low consumption of fruit and vegetables [11–14] and high consumption of foods with added sugar [15, 16], compared to those in families with high SES. Studies have also shown that females tend to consume fruit and vegetables more frequently [11, 17] and consume soft drinks less frequently than males [18, 19].

In 2007, the Norwegian government launched the Norwegian School Fruit Scheme (NSFS) to promote and increase the consumption of fruit and vegetables among children and adolescents. The NSFS provided students in all secondary schools (grades 8–10) and all combined schools (grades 1–10) with a free (not parent-funded) piece of fruit or vegetable every school day, usually during lunchtime. Lasting a total of seven years, this is possibly the most comprehensive and costly initiative targeting a specific dietary behaviour among children and adolescents in Norway.

Among children, school-based intervention studies aiming to increase fruit and/or vegetable consumption have shown promising effects during their operation [20, 21]. In Norway, two separate evaluation studies of the NSFS have shown that the program increased fruit consumption among children, regardless of gender and SES [22, 23]. Additionally, a pilot version of the NSFS seemed to reduce the consumption of snacks among children while operating [24, 25]. However, the effect of the NSFS on Norwegian adolescents’ dietary habits is yet to be evaluated. In addition, the number of studies reporting the effect of interventions providing free fruit and vegetables to adolescents is limited.

The primary objective of this study was to evaluate the effects of the NSFS on consumption of fruit, vegetables, potato chips, candy, SSB and artificially sweetened beverages (ASB) among adolescents, and whether the effect varied according to SES (educational intentions), gender or grade. The secondary objective was to examine changes in dietary consumption of fruit, vegetables, potato chips, candy and SSB among adolescents between 1995 and 2008, and how any gender and SES differences developed in this period. The third objective was to examine changes in dietary habits in NSFS schools and control schools between 1995 and 2008, to determine whether dietary trends developed differently according to school type.

## Methods

### Study design and study sample

We used cross-sectional data from the Young-HUNT study collected between 1995 and 1997 (Young-HUNT1) and between 2006 and 008 (Young-HUNT3). The Young-HUNT study is the adolescent (13–19 years) arm of the Nord-Trøndelag Health (HUNT) study, a longitudinal health study in Norway [26]. It was designed to cover several topics related to major public health issues, including respiratory and allergic disease, subjective health problems, eating habits, and overweight and obesity. Schools have been the main study site in the Young-HUNT surveys, with all 66 schools in the county of Nord-Trøndelag participating. All adolescents, and parents of adolescents under the age of 16, gave written consent to participate. For practical reasons, data were collected in one school at a time and within one municipality, before moving on to the next. In the present study we only included grades 8–10 in junior high schools, where the response rate was 92% (*n* = 4596) in 1995–1997 and 82% (*n* = 4615) in 2006–2008 [26].

To evaluate the NSFS, the study used a natural experimental design characterised by exposure to the intervention having not been manipulated by the researcher [27]. We exploited data collection for the Young-HUNT3 survey being conducted in the same period as NSFS implementation. The NSFS was implemented in all secondary schools (grades 8–10) and combined schools (grades 1–10) during autumn 2007. On each school day, students who were part of the NSFS were offered one kind of fruit or vegetable. Apples, pears, bananas, oranges, clementines, kiwis, carrots and nectarines were most frequently offered.

We defined 1 September 2007 as the start date of the NSFS initiative. The date when participants answered the study questionnaire was used to identify affiliation to either the intervention group or the control group. Adolescents in grades 8–10 who answered the Young-HUNT3 questionnaire after the implementation of the program were classified as the intervention group (September 2007 to July 2008, *n* = 1892) and those who answered the questionnaire before the implementation (Spring 2006 to 31 August 2007, *n* = 2855) as the control group.

We aggregated data from the two Young-HUNT surveys to explore possible changes in dietary habits between 1995 and 2008 according to educational intentions, gender and school type. During the period between 1995 to 2008 some schools had been closed (*n* = 9) and new schools (n = 9) had been opened. To explore the change over time, we only included adolescents attending schools (*n* = 35) operating in both surveys. Thus, 4113 and 4137 were included from Young-HUNT3 and Young-HUNT1, respectively.

We further explored secular changes in dietary habits according to schools’ NSFS status. Intervention schools and control schools within Young-HUNT3 (2008) were compared to the same schools within Young-HUNT1 (1997). Thus, within the young-HUNT1 sample, we defined the group “intervention schools” (*n* = 1791) and the group “control schools” (*n* = 2346). The majority of schools in 2006–08 either had pupils in the intervention group *or* the control group. However, five schools in the 2006–08 study had pupils in both groups, but most students in these schools were in the control group (only 20 students were part of the intervention group). For secular analysis according to NSFS status, these five schools were categorised as “control schools” within Young-HUNT1, as most students in these schools were part of the control group within Young-HUNT3.

### Measurements

#### Dietary consumption

Adolescents’ dietary habits were measured by the same food frequency questionnaire (FFQ) used in Young-HUNT1 and Young-HUNT3. Consumption of fruit, vegetables, potato chips and candy were measured by the question: “How often do you eat the items listed below?” The reply options were: several times a day, once a day, every week but not every day, less than once a week and never. Consumption of SSB and ASB (only included in Young-HUNT3) was measured by the wording: “How often do you drink the items listed below?” The reply options were: seldom/never, 1–6 glasses a week, 1 glass a day, 2–3 glasses a day and 4 or more glasses a day. Consumption was dichotomised into daily consumption (more than once a day and once a day) and less than daily consumption (every week but not every day, seldom and never) of fruit, vegetables, potato chips, candy, SSB and ASB. Participants completed the questionnaire during a school hour, in an exam-like setting.

The dietary questions in the Young-HUNT surveys were based on the FFQ used in the Health Behaviour in School-aged Children (HBSC) study. The validity of the HBSC FFQ has previously been reported for adolescents [28]. Reliability analysis (interval: 6–15 days) resulted in Spearman correlation values ranging from 0.45 to 0.82 among 11–12-year-olds and from 0.57 to 0.78 among 13–14-year-olds, with an overall mean correlation of 0.70 and 0.67, respectively.

Spearman correlation between the FFQ and the seven-day food diary (reference method) was 0.34 for fruit, 0.48 for vegetables, 0.46 for SSB, 0.15 for ASB, 0.10 for potato chips and 0.25 for candy. Comparison between the two methods showed that the FFQ overestimated all food items except SSB and potato chips.

#### Sociodemographic variables

Adolescents’ own educational intention was the best proxy for SES available in the Young-HUNT dataset. A previously published study used both educational intentions and parental education as proxies for SES, and the results showed almost identical results when comparing the association between beverage consumption and the two proxies [29]. Therefore, we have reason to believe that educational intention is an acceptable proxy for SES when assessing dietary consumption among adolescents.

Adolescents’ educational intention was measured by the question: “What plans for further education do you have?” The reply options in Young-HUNT3 were: university or university college four years or more, university or university college less than four years, other vocational education, no plans, or don’t know. Young-HUNT1 included two additional reply options: high school general education and secondary vocational education (no plans was not listed as a reply option). As it was possible to choose more than one reply option, we used the highest level of education registered by the participant. The variable was further dichotomised into “higher educational intentions” (college or university) and “lower educational intentions”.

The county of Nord-Trøndelag included 24 municipalities in which six of the municipalities included villages with city status. The variable “municipality urbanity” used in the present study had two categories: rural and urban. Being categorised as urban reflected that the participant lived in one of the six municipalities that included villages with city status. In Young-HUNT1, participants reported their own gender, and which grade they attended. In Young-HUNT3, however, this information was collected by using participants’ personal identification numbers. In both surveys, sociodemographic data (urbanity of the municipality and which school they attended) were registered by using personal identification number and linking this to the school register and national population register.

### Statistics

#### Evaluation of the NSFS (using data from young-HUNT3)

Descriptive statistics were presented using independent t-tests for continuous variables and chi-square for categorical variables. To evaluate the NSFS, the outcomes (fruit, vegetables, candy, SSB, ASB and potato chips) were analysed separately by multilevel logistic regression models. Schools were included as a random intercept. The models included the covariates gender, grade, educational intentions and municipality urbanity. We tested a priori for interactions between adolescents’ exposure to the NSFS and (i) gender, (ii) educational intentions and (iii) grade level.

#### Change of dietary patterns over time in relation to SES, gender and school type

We investigated change in dietary habits (fruit, vegetables, candy, potato chips and SSB) between 1995 (Young-HUNT1) and 2008 (Young-HUNT3) by multilevel logistic regression. School was included as a random intercept. The six outcomes were analysed separately, by two models. To examine possible changes in dietary patterns, a binary variable “time” was constructed (1995 [HUNT1] =0; 2008 [HUNT3] =1). In model 1, we analysed the main effect of time, educational intentions, gender, grade and municipality urbanity. In model 2, we included the interactions between time and gender and time and educational intentions.

Secondly, to explore secular trends according to school type, an additional analysis was conducted. The binary variable “school type” was constructed, in which schools in the intervention group in Young-HUNT3 and the same schools in the Young-HUNT1 (intervention schools) were treated as one group “NSFS schools” (coded 1); the schools in the control group in Young-HUNT3 and the same schools in Young-HUNT1 (control schools) were treated as another group “control schools” (coded 0). To explore whether the secular change was different between NSFS schools and control schools from 1995 to 2008, the interaction term between school type and time (HUNT1 vs HUNT3) was tested in a multilevel logistic regression model with a random intercept for school, for all six dietary outcomes. The models included the covariates gender, grade, educational intentions and urbanity. If the interaction term (school type* time) was significant for a dietary outcome, stratification was performed by school type.

In all analysis, we used *p* < 0.05 to indicate statistically significant associations. For interaction terms *p* < 0.1 was used, as interaction terms are a multiplication of two variables that include measurement error [30]. All models reported odds ratio (OR) with a robust 95% confidence interval (CI). All statistical analyses were conducted using Stata 15.1.

## Results

### Characteristics of the study sample

The proportion of participants who consumed fruit daily was higher in the intervention group (67% vs 57%, *p* < 0.001) than the control group. In the intervention group the mean age was lower (14.5 vs 14.7 years, p < 0.001), and a lower percentage lived in an urban area compared to the control group (63% vs 70%, p < 0.001). The distribution of participants according to grade level varied between the intervention group and the control group: 33% vs 36% attended grade 8, 33% vs 34% attended grade 9, and 30% vs 33% addended grade 10, respectively (*p* = 0.026).

A higher percentage of Young-HUNT3 participants consumed fruit (61% vs 50%, *p* < 0.001), vegetables (52% vs 41%, *p* < 0.001), potato chips (6% vs 4%, p < 0.001) and SSB daily (29% vs 17%, p < 0.001) compared to Young-HUNT1 participants. Furthermore, a higher percentage of Young-HUNT3 participants had higher educational intentions (34% vs 15%, *p* < 0.001), lived in an urban municipality (63% vs 60%, *p* = 0.013) and had a lower mean age (14.5 years vs 14.6 years, p < 0.001) compared to Young-HUNT1 participants. The distribution of participants according to grade level was different in Young-HUNT1 and Young-HUNT3: 32% vs 34% attended 8th grade and 35% vs 32% addended 10th grade, respectively (*p* = 0.005).

Within Young-HUNT1, a significantly higher number of participants in the “intervention schools 1995” group had higher educational intentions (16% vs 14%, *p* = 0.012), lived in an urban municipality (62% vs 58%, *p* = 0.006) and consumed SSB (19% vs 16%, p = 0.012) compared to participants in the “control schools 1995” group (Table 1). The mean age (14.6 vs 14.7, *p* = 0.029) was significantly different in the groups.

**Table 1:** Characteristics of study population within Young-HUNT1; control schools and intervention schools, Young-HUNT1 and Young-HUNT3 and within Young-HUNT3; intervention group and control group

	Young-HUNT1***			Young-HUNT1 vs Young-HUNT3			Young-HUNT 3*		
	Control schools (*n* = 2346)	Intervention schools (*n* = 1791)	*p*-value	Young-HUNT1 (N = 4137)	Young-HUNT3 (N = 4113)	*p*-value	Control group (N = 2855)	Intervention group (N = 1892)	*p*-value
Age (mean)	14.6	14.7	0.029	14.6	14.5	< 0.001	14.7	14.5	< 0.001
	%	%		%	%		%	%	
Sex (female)	50	52	0.175	51	50	0.524	49	50	0.740
Educational intentions (higher)	14	16	0.012	15	34	< 0.001	34	35	0.680
Municipality urbanity (urban)	58	62	0.006	60	63	0.013	70	63	< 0.001
Grade junior high			0.399			0.005			0.026
8 th grade	32	31		32	34		33	36	
9 th grade	34	33		34	34		34	33	
10 th grade	34	36		35	32		33	30	
Dietary consumption (daily)									
Fruit	50	51	0.504	50	61	< 0.001	57	67	< 0.001
Vegetables	40	43	0.080	41	52	< 0.001	51	53	0.088
Potato chips	4	4	0.649	4	6	< 0.001	7	6	0.353
Candy	12	13	0.502	13	12	0.555	13	12	0.517
Sugar-sweetened beverages	16	19	0.012	17	29	< 0.001	29	28	0.469
Artificially sweetened beverages**	n.a	n.a	n.a	n.a	19	n.a	19	19	0.806

*In Young-HUNT3, participants who answered the questionnaire before the 1. Of September 2007 was defined as the control group and participants who answered after 1. September 2007 as the intervention group

N.a (not applicable). **Consumption of artificially sweetened beverages was not measured in the Young-HUNT1 survey

***Young-HUNT1 control schools and intervention schools grouping was based on schools by intervention and control in Young-HUNT3

### The NSFS effect on dietary behaviour (using data from young-HUNT3)

The intervention group showed increased odds of daily fruit consumption (aOR = 1.75, 95% CI = 1.25–2.43) compared to the control group (Table 2). We did not observe any effect modification between exposure to the NSFS and i) gender (*p* = 0.548), ii) grade (*p* = 0.101) or iii) educational intentions (*p* = 0.554) for the outcome of fruit (analysis not shown). No significant differences between the intervention and control group were found for vegetables (aOR = 1.09, 95% CI = 0.92–1.29), potato chips (aOR = 0.95, 95% CI = 0.70–1.28), candy (aOR = 0.91, 95% CI = 0.70–1.18), SSB (aOR = 1.10, 95% CI = 0.87–1.39) and ASB (aOR = 0.99, 95% CI = 0.82–1.21).

**Table 2:** Odds ratio (OR) of daily consumption of fruit, vegetables, potato chips, candy, sugar-sweetened beverages, artificially sweetened beverages, intervention vs control in Young-HUNT3 (*n* = 4747)

Outcome	Unadjusted analysis (odds ratio, 95% CI)	*Adjusted analysis (odds ratio, 95% CI)	*p*-value (adjusted analysis) **
Fruit	1.77 (1.25–2.51)	1.75 (1.25–2.43)	0.001
Vegetable	1.13 (0.96–1.35)	1.09 (0.92–1.29)	0.289
Potato chips	0.94 (0.70–1.25)	0.95 (0.70–1.28)	0.740
Candy	0.92 (0.72–1.17)	0.91 (0.70–1.18)	0.498
Sugar-sweetened beverages	1.06 (0.85–1.33)	1.10 (0.87–1.39)	0.419
Artificially sweetened beverages	1.02 (0.85–1.21)	0.99 (0.82–1.21)	0.994

*Adjusted for covariates grade, gender, educational intentions and urbanity in municipality

The reference group for all outcomes was the control group

### Change of dietary patterns over time in relation to SES and gender

Firstly, we interpret the models without interactions (Model 1, Table 3). Compared to study participants in 1995, those participating in 2008 showed increased odds of daily consumption of fruit (aOR = 1.48, 95% CI = 1.28–1.71), vegetables (OR = 1.41, 95% CI = 1.28–1.53), potato chips (aOR = 1.60, 95% CI = 1.26–2.04) and SSB (OR = 2.02, 95% CI = 1.66–2.45). No difference was observed for consumption of candy (aOR = 0.97, 95% CI = 0.83–1.12).

**Table 3:** Development over time in relation to planned education and gender (only schools with participants in both surveys)

	Daily consumption of fruit						Daily consumption of vegetables					
	Model 1			Model 2			Model 1			Model 2		
	aOR	95% CI	p-value	aOR	95% CI	*p*-value	aOR	95% CI	*p*-value	aOR	95% CI	*p*-value
HUNT 3 vs. HUNT1	1.48	1.28–1.71	< 0.001	1.60	1.19–1.98	< 0.001	1.41	1.28–1.53	< 0.001	1.42	1.23–1.65	< 0.001
Educational intentions	1.45	1.28–1.62	< 0.001	1.42	1.20–1.68	< 0.001	1.51	1.35–1.68	< 0.001	1.35	1.14–1.60	< 0.001
Gender	0.68	0.62–0.75	< 0.001	0.74	0.65–0.85	< 0.001	0.82	0.74–0.91	< 0.001	0.86	0.76–0.98	0.025
Grade												
9 th grade	0.84	0.72–0.97	0.022	0.84	0.72–0.97	0.022	0.79	0.69–0.91	0.001	0.80	0.69–0.91	0.001
10 th grade	0.68	0.58–0.79	< 0.001	0.69	0.58–0.79	< 0.001	0.74	0.63–0.87	< 0.001	0.74	0.63–0.88	< 0.001
Urbanity	1.15	0.94–1.42	0.158	1.15	0.95–1.41	0.162	1.12	0.97–1.29	0.106	1.12	0.97–1.29	0.117
Constant	1.33	1.14–1.55	< 0.001	1.28	1.07–1.53	0.005	0.84	0.97–0.96	0.014	0.83	0.70–0.97	0.021
Time x educational intentions				1.02	0.87–1.20	0.786				1.18	0.97–1.43	0.094
Time x gender				0.85	0.68–1.06	0.144				0.90	0.76–1.06	0.231
	Daily consumption of potato chips						Daily consumption of sugar-sweetened beverages					
	Model 1			Model 2			Model 1			Model 2		
	aOR	95% CI	p-value	aOR	95% CI	*p*-value	aOR	95% CI	*p*-value	aOR	95% CI	*p*-value
HUNT 3 vs. HUNT1	1.60	1.26–2.04	< 0.001	1.10	0.73–1.68	0.623	2.02	1.66–2.45	< 0.001	1.92	1.51–2.43	< 0.001
Educational intentions	0.84	0.68–1.04	0.113	0.63	0.44–0.92	0.015	0.78	0.67–0.92	0.002	0.81	0.67–0.95	0.014
Gender	2.30	1.89–2.80	< 0.001	1.76	1.32–2.35	< 0.001	2.19	1.90–2.53	< 0.001	2.07	1.73–2.47	< 0.001
Grade												
9 th grade	0.91	0.72–1.15	0.443	0.91	0.72–1.15	0.447	1.20	1.05–1.38	0.009	1.20	1.04–1.38	0.009
10 th grade	0.84	0.65–1.08	0.181	0.84	0.65–1.08	0.175	1.44	1.26–1.64	< 0.001	1.44	1.27–1.64	< 0.001
Urbanity	0.71	0.52–0.95	0.022	0.70	0.52–0.94	0.021	0.73	0.59–0.89	0.002	0.73	0.59–0.89	0.002
Constant	0.03	0.02–0.03	< 0.001	0.04	0.03–0.05	< 0.001	0.14	0.11–0.15	< 0.001	0.13	0.11–0.16	< 0.001
Time x educational intentions				1.45	0.97–2.17	0.067				0.96	0.73–1.28	0.822
Time x gender				1.57	1.06–2.34	0.024				1.10	0.91–1.34	0.312
	Daily consumption of candy											
	Model 1			Model 2								
	aOR	95% CI	*p*-value	aOR	95% CI	*p*-value						
HUNT 3 vs. HUNT1	0.97	0.83–1.12	0.664	0.92	0.72–1.17	0.501						
Educational intentions	0.87	0.76–0.99	0.044	0.90	0.70–1.16	0.449						
Gender	1.48	1.32–1.65	< 0.001	1.40	1.19–1.64	< 0.001						
Grade												
9 th grade	1.13	0.92–1.38	0.193	1.13	0.94–1.37	0.192						
10 th grade	1.30	1.04–1.62	0.020	1.30	1.04–1.62	0.021						
Urbanity	0.88	0.69–1.15	0.376	0.89	0.69–1.15	0.383						
Constant	0.11	0.08–0.13	< 0.001	0.10	0.08–0.14	< 0.001						
Time x educational intentions				0.93	0.69–1.26	0.673						
Time x gender				1.11	1.19–1.64	0.673						

The reference category for gender: female, Time: Young-HUNT1, Educational intentions: lower educational intentions, grade: 8th grade, Urbanity: urban area

School was included as a random intercept

The analysis revealed that those with higher educational intentions were more likely to report daily consumption of fruit (aOR = 1.45, 95% CI = 1.28–1.62) and vegetables (aOR = 1.51, 95% CI = 1.35–1.68) and less likely to report daily consumption of SSB (aOR = 0.78, 95% CI = 0.67–0.92) and candy (aOR = 0.87, 95% CI = 0.76–0.99). Gender differences were found for all dietary outcomes. Males were less likely to eat fruit (aOR = 0.68, 95% CI = 0.62–0.75) and vegetables (aOR = 0.82, 95% CI = 0.74–0.91), and more likely to consume potato chips (aOR = 2.30, 95% CI = 1.89–2.80), SSB (aOR = 2.19, 95% CI = 1.90–2.53) and candy (aOR = 1.48, 95% CI = 0.76–0.99).

Secondly, we interpret the models with interactions (Model 2, Table 3). The interaction between educational intentions and time for the outcomes of fruit, SSB and candy was not significant. The interaction between educational intentions and time was significant for vegetables (aOR = 1.18, 95% CI = 0.97–1.43) and potato chips (aOR = 1.45, 95% CI = 0.97–2.17). Between 1995 and 2008 the interaction between gender and food habits was not significant, except for daily consumption of potato chips (aOR = 1.57, 95% CI = 1.06–2.34).

### Change of dietary patterns over time in relation to school type

The results from the analysis exploring change in dietary patterns according to school type revealed that the interaction between school type (NSFS schools vs control schools) and time (1995 vs 2008) was only significant for fruit (*p* = 0.014, adjusted model, analysis not shown). As there was a significant interaction between school type and time for the outcome of fruit, stratification was done by school type (see Additional file 1 for stratified analysis).

Stratification by school type revealed that adolescents at NSFS schools had an increased odds of 1.82 (95% CI = 1.38–2.38) of daily consumption of fruit in 2008 compared to adolescents in the same schools in 1995, while adolescents at control schools had an increased odds of 1.26 (95% CI = 1.07–1.47) of daily consumption of fruit in 2008 compared to the same schools in 1995 (Additional file 1: Table S1). No effect modification was found for gender or educational intentions in the stratified model (not included in the model in Additional file 1).

## Discussion

### The NSFS effect on dietary behaviour

The results implied an overall positive effect of the NSFS on adolescents’ fruit consumption, regardless of gender, educational intentions or grade level. As most school fruit and vegetable interventions have targeted children, a limited number of comparable studies have investigated the effects of such interventions among adolescents [20, 21]. In line with our results, a pilot study conducted in the USA that provided free fruit and vegetables for one school year indicated that adolescents (grades 8 and 10) increased their overall fruit consumption but not vegetable consumption [31]. However, that study did not use a control group, limiting the ability to attribute the increased fruit consumption to the program. Also, in line with our present results, previous evaluation studies of the NSFS have also confirmed that the program increased fruit but not vegetable consumption among children, regardless of gender and SES [22, 23].

Contrary to our findings, results from previously published studies targeting children have shown that free fruit and vegetable interventions also seem to reduce the consumption of snacks during the intervention [24, 25]. Differences in effect on snack consumption in studies evaluating the effectiveness of fruit and vegetable interventions may partly be explained by the use of different measures of “snack” consumption, and how long participants were part of the intervention. In our analysis, we evaluated each food item separately, not merging serval items into one “snacks” category, which was done in the studies that found reduced consumption of snacks [24, 25]. Further, participants in our sample had been part of the NSFS for an average 4.3 months (95% CI = 4.2–4.4) when answering the questionnaire (range = 0.1–9.7 months), while the children in the two aforementioned studies had been part of a school fruit and vegetable intervention for approximately one year [24, 25].

### Change of dietary patterns over time in relation to SES, gender and school type

Overall, we found an increased frequency of daily consumption of fruit and vegetables between 1995 and 2008, which is in line with other studies, from both Norway [12, 18] and other European countries [7]. The present study found that adolescents increased their frequency of daily consumption of SSB between 1995 and 2008, which is in contrast with previous findings from Norway, among both children and adolescents [15, 18]. In contrast with a previous study, which showed that the frequency of SSB consumption decreased among adolescents in Norway between 2001 and 2009 [18], our results indicated that the adolescent population in Nord-Trøndelag county increased their frequency of daily SSB consumption between 1995 and 2008. This finding was surprising as the county has been considered fairly representative of Norway as a whole [26].

The present study identified both positive and negative changes in dietary trends among adolescents according to SES and gender. Our findings suggest that socioeconomic inequalities in fruit consumption were stable between 1995 and 2008, which has previously been confirmed by other studies from the Nordic region [13]. Conversely, our results indicated that the impact of educational intentions increased for vegetable consumption between 1995 and 2008. Another study among Norwegian adolescents, however, did not reveal any differences in vegetable consumption according to SES between 2001 and 2009 [18].

In a Norwegian context, this is to our knowledge the first study to measure change in potato chips consumption according to SES and gender among adolescents. For potato chips, our results indicated that the difference in consumption between those with higher and lower educational intentions decreased between 1995 and 2008. Further, our results indicated that males increased their consumption of potato chips compared to females between 1995 and 2008. Contrary to our results, Scotland observed no changes in potato chips consumption according to SES, and their results indicated that males reduced their consumption in approximately the same time period [32]. Both aforementioned studies used the family affluence scale as an indicator for SES, which may explain differences in result regarding change of vegetable and potato chips consumption in relation to SES over time [18, 32].

When assessing socioeconomic disparities, the SES indicator must be kept in mind, as the use of different indicators might yield different results. There is no single superior indicator of SES, as each measures different aspects and can be more or less relevant to different outcomes at different life stages [33]. The present study used educational intentions as an indicator of SES, which has previously been used as an indicator among this age group [29].

Secularly, the frequency of fruit consumption increased between 1995 and 2008 (aOR = 1.48, Table 3) and this increased frequency differed by school type (aOR = 1.82 vs aOR = 1.26, Additional file 1: Table S1). This indicates that the secular effect of the NSFS, where adolescents were exposed for a maximum of 11 months, overwhelms the secular increased frequency of fruit in the previous decade. Contrary to our findings, a cross-sectional study among a representative sample of Norwegian adolescents aged 11, 13, 15 and 16 years found an increase in fruit consumption between 2001 and 2005, but no further improvement between 2005 and 2009 [18]. Although that study did not aim to evaluate the NSFS, its results indicated that the NSFS did not have an effect on adolescents’ fruit consumption. However, it used a different sample and assessed dietary changes between 2001 and 2009.

### Strengths and limitations

A key strength of the present study was the use of the large population-based Young-HUNT study, conducted in the county of Nord-Trøndelag, which has been considered representative of Norway regarding demographic factors though it lacks larger cities [26]. Our results may be representative of adolescents attending schools in other Norwegian counties, and also other countries with similar demographic and sociodemographic distribution. However, the results may not be generalisable for adolescents living in large cities.

Another strength was our natural experimental design, which enabled evaluation of a past public health initiative according to SES. Our results add to the body of literature which has shown that “upstream” interventions, such as free fruit and vegetables at school, are more likely to be effective among individuals regardless of SES [34].

A limitation of our study was that some significant differences existed between the intervention group and the control group. Within Young-HUNT3, a higher percentage of the control group lived in an urban municipality compared to the intervention group. This was most likely due to the fact that the data collection was completed in one municipality before moving on to the next. Further, it was not possible to obtain the exact date of implementation of the NSFS at different schools; therefore, we defined 1 September as the start date. However, assuming an implementation date prior to the actual implementation date would most likely lead to an underestimation of the NSFS effect. One key limitation of this study was that the participants in the intervention group had received different exposure to the NSFS (ranging from 0.1 to 9.7 months), which may also have led to an underestimation of the effect of the initiative.

Another important limitation was that we could not conduct a more comprehensive evaluation of the NSFS and dietary patterns over time. The questionnaire used only measured the frequency of certain food items [26]. We recognise that information on adolescents’ actual food consumption and portion sizes would have provided a more detailed picture. In addition, the data was self-reported, which is known to be prone to bias.

## Conclusion

Our results suggested that the NSFS contributed to an increased frequency of daily fruit consumption among adolescents, regardless of gender and educational intentions and grade level. Moreover, results indicated an overall increased frequency of fruit, vegetable, SSB and potato chips consumption between 1995 and 2008. However, the secular effects for fruit varied by school type, which indicates that the secular effect of the NSFS overwhelms the secular increased frequency of fruit in the previous decade. Between 1995 and 2008, the socioeconomic gap in vegetable consumption increased and the socioeconomic gap in potato chips consumption decreased. Finally, the results showed an increased frequency of potato chips consumption among males compared to females between 1995 and 2008.

Free school fruit schemes thus seem to be an effective approach to increase fruit consumption among all Norwegian adolescents. Similar efforts should be used to increase vegetable consumption. The effect of reducing the availability of unhealthy food items and beverages, and reducing socioeconomic differences in dietary habits, should also be further investigated in future studies.

## Supplementary information

**Additional file 1.** Odds ratio for daily consumption of fruit stratified by group. (DOCX 22 kb)

## Data Availability

The data that support the findings of this study are available from HUNT Research Centre, but restrictions apply to the availability of these data, which were used under license for the current study, and so are not publicly available.

## Abbreviations

HUNT: the Nord-Trøndelag Health Study
NSFS: Norwegian School Fruit Scheme
