# Supplementary material for: Time trends (1995–2008) in dietary habits among adolescents in relation to the Norwegian school fruit scheme: the HUNT study
Source: Nutr J. 2019 Nov 20;18:77. doi: 10.1186/s12937-019-0501-z (PMC6868806; doi:10.1186/s12937-019-0501-z)
Supplement: Supplementary file 1 — Additional file 1. Odds ratio for daily consumption of fruit stratified by group. (DOCX 22 kb) [file 12937_2019_501_MOESM1_ESM.docx]

Additional Table 1, Odds ratio for daily consumption of fruit stratified by School type.

|  | Daily consumption of fruit | | | | | |
| --- | --- | --- | --- | --- | --- | --- |
|  | **NSFS schools** | | | **Control schools** | | |
|  | OR | 95 % CI | p-value | OR | 95% CI | p-value |
| Time (HUNT3 vs HUNT1) | 1.82 | 1.38-2.38 | <0.001 | 1.26 | 1.07-1.47 | 0.005 |
| Educational intentions (higher) | 1.54 | 1.29-1.85 | <0.001 | 1.38 | 1.21-1.59 | <0.001 |
| Gender (males) | 0.73 | 0.65-0.82 | <0.001 | 0.64 | 0.57-0.74 | <0.001 |
| Grade |  |  |  |  |  |  |
| 9 th | 0.83 | 0.62-1.10 | 0.198 | 0.85 | 0.73-1.01 | 0.075 |
| 10 th | 0.76 | 0.58-0.99 | 0.044 | 0.63 | 0.53-0.76 | <0.001 |
| Urbanity (rural) | 1.45 | 1.07-1.96 | 0.014 | 0.99 | 0.78-1.27 | 0.990 |
| constant | 1.11 | 0.88-1.41 | 0.348 | 1.49 | 1.23-1.81 | <0.001 |

*Adjusted for grade, urbanity in municipality, gender and educational intentions. School included as a random intercept. NSFS schools includes HUNT1 intervention schools and Young-HUNT3 intervention group. Control schools includes control schools in YoungHUNT1 and control group in Young-HUNT3.
